# Hypomania and saccadic changes in Parkinson’s disease: influence of D2 and D3 dopaminergic signalling

**DOI:** 10.1038/s41531-019-0107-3

**Published:** 2020-01-17

**Authors:** Esther A. Pelzer, Barbara Dillenburger, Sophie Grundmann, Vladimir Iliaev, Sophie Aschenberg, Corina Melzer, Martin Hess, Gereon R. Fink, Carsten Eggers, Marc Tittgemeyer, Lars Timmermann

**Affiliations:** 1 Max-Planck Institute for Metabolism Research, Gleulerstr. 50, 50931 Cologne, Germany; 2 Department of Neurology, University Hospital Cologne, Kerpenerstr. 62, 50931 Cologne, Germany; 3 Cognitive Neuroscience, Institute of Neuroscience and Medicine (INM-3), Research Center Jülich Cologne, 52428 Jülich, Germany; 4 University Hospital Marburg, Baldingerstrasse, 35043 Marburg, Germany; 5 Cologne Cluster of Excellence in Cellular Stress and Aging Associated Disease (CECAD), Cologne, Germany

**Keywords:** Clinical genetics, Parkinson's disease, Oculomotor system

## Abstract

In order to understand the influence of two dopaminergic signalling pathways, TaqIA rs1800497 (influencing striatal D2 receptor density) and *Ser9Gly* rs6280 (influencing the striatal D3 dopamine-binding affinity), on saccade generation and psychiatric comorbidities in Parkinson’s disease, this study aimed to investigate the association of saccadic performance in hypomanic or impulsive behaviour in parkinsonian patients; besides we questioned whether variants of D2 (A1+/A1−) and D3 (B1+/B1−) receptor polymorphism influence saccadic parameters differently, and if clinical parameters or brain connectivity changes modulate this association in the nigro-caudatal and nigro-collicular tract. Initially, patients and controls were compared regarding saccadic performance and differed in the parameter duration in memory-guided saccades (MGS) and visually guided saccades (VGS) trials (*p* < 0.0001) and in the MGS trial (*p* < 0.03). We were able to find associations between hypomanic behaviour (HPS) and saccade parameters (duration, latency, gain and amplitude) for both conditions [MGS (*p* = 0.036); VGS (*p* = 0.033)], but not for impulsive behaviour. For the A1 variant duration was significantly associated with HPS [VGS (*p* = 0.024); MGS (*p* = 0.033)]. In patients with the B1 variant, HPS scores were more consistently associated with duration [VGS (*p* = 0.005); MGS (*p* = 0.015), latency [VGS (*p* = 0.022)]] and amplitude [MGS (*p* = 0.006); VGS (*p* = 0.005)]. The mediation analysis only revealed a significant indirect effect for amplitude in the MGS modality for the variable UPDRS-ON (*p* < 0.05). All other clinical scales and brain connectivity parameters were not associated with behavioural traits. Collectively, our findings stress the role of striatal D2 and D3 signalling mechanisms in saccade generation and suggest that saccadic performance is associated with the clinical psychiatric state in Parkinson’s disease.

## Introduction

Changes in saccadic performance are strongly associated with dopaminergic modulation of the reward system, as shown in the human species^1^ and in the monkey.^2–5^ Diverse anatomical regions participate in the control of saccades subsuming cortical (e.g., the dorsolateral prefrontal cortex and the frontal eye field) as well as subcortical regions [e.g., substantia nigra (SN), superior colliculus (SC) and caudate nucleus (CD)], the brainstem and the cerebellum [for overview in the human species and the monkey see Leigh and Kennard^6^]. Especially the reticular part of the SN plays a key role in in the subcortical control of monkeys’ eye movement.^7^ Here, the CD sends inhibitory projections to the reticulate part of the SN, and the SN again projects to SC with inhibitory output, sub-cortically controlling the motoric saccadic output.^8^ Interestingly, context dependency of saccadic activity in this network suggests that this activity is not only a result of corollary signals from oculomotor centres, but contributes to sensory–motor transformation and functions of the prefrontal cortex as shown for both species.^9–11^ In general, various parameters of saccadic performance such as latency, amplitude, velocity and gain are frequently affected in PD patients, although with quite heterogenic and inconsistent findings.^12^ Moreover, parkinsonian patients often show a stronger affection of (internally generated) memory-guided saccades (MGS) than of (externally driven) reflexive (visually guided saccades (VGS)) saccades.^13^ Hence, the oculomotor system connects the visual perception with motor reaction, resulting in behavioural changes due to external stimuli and offers insights into disease pathology of the underlying movement disorders, such as Parkinson’s disease (PD).

PD is a progressive neurodegenerative disease with pathological deposits in various systems of the brain.^14^ It is becoming increasingly clear that the disease relates not only to a deficiency of the motor system, but also includes non-motor categories like hypomanic symptoms or impulsive behaviour.^15,16^ Thereby, recent studies suggest a prevalence of >10% for impulsive behavioural phenotypes and for HPS in PD.^17,18^ These symptoms, however, are highly relevant in daily life with high impact on social interactions of both, patients and their caregivers. HPS,^19^ for instance, severely affects and often even determines the clinical outcome of dopamine replacement therapy and deep brain stimulation in PD patients.^20^ Yet until now, no safe clinical predictors exist to exclude patients from therapies that yield to an exacerbation of HPS due to the fact that both impulsive and HPS are not overt behaviours that are formally or clinically diagnosed but are rather traits that can be revealed by questionnaires. In line with this, impulsive behaviour,^21^ on the other hand, might not necessarily relate to dysfunctional states.^22^ From an evolutionary point of view, in a competing situation, impulsive behaviour can be advantageous to other “rivals” due to a faster reaction onto, e.g., time-sensitive rewards.^23^

Though hypomanic as well as impulsive behaviour have been linked to saccadic performance changes in general, these changes are heterogeneous, inconsistent and under-studied.^24^ Whether and to what extent parameters of saccadic performance are altered in PD and differentially relate to these behavioural phenotypes are not clear. The answer to this question, however, could lead to an interesting biomarker by elucidating putative treatment side effects in an early stage.

Dopamine shapes a wide variety of psychomotor functions. To that end, the expression of hypomanic and impulsive behaviour is closely linked to D2 and D3 receptor signalling. Genetic variants that influence DA signalling and shape the characteristics of these behavioural phenotypes provide interesting means to further investigate this relation. Here, dopaminergic gene variations through the *TaqIA* restriction fragment length polymorphism [rs180049^25,26^] and by the *Ser9Gly* gene polymorphism [rs6280^27^] may be considered in driving D2R and D3R signalling mechanisms relating with hypomanic and impulsive behaviour, respectively. Interestingly, an association of the D2 and D3 polymorphisms as disease-causing variants have been reported for PD.^28^

The TaqIA single-nucleotid polymorphism (SNP) is associated with a mutation producing a single amino acid change within the substrate-binding domain of the ankyrin repeat and kinase domain-containing 1 (*ANKK1*) protein,^29^ and is in linkage disequilibrium with the D2 receptor locus.^30^ In humans, the A1 allele of the (*ANKK1*) TaqIA (rs1800497) SNP has been associated with reduced striatal dopaminergic D2 receptor availability.^31–33^ Healthy individuals who carry the A1 allele (A1+, risk allele), compared with those who do not (A1−, wild type), show diminished striatal D2 receptor density^34^ and reduced glucose metabolism in regions involved in reward processing and receiving dopaminergic innervations.^35^ Functionally, striatal medium spiny neurons of the indirect pathway primarily express dopamine D2 receptors and help to suppress cortical patterns that encode maladaptive or non-rewarding actions.^36,37^ An affection of these striatal neurons can result in impulsive behaviour,^38^ and dopamine-relevant polymorphisms, especially for the D2 receptor gene, are associated with an increased risk to develop a (hypo-)mania in non-Parkinsonian humans.^26^

The most frequently studied variation of a D3 receptor gene is the Ser9Gly polymorphism [rs6280^39^], which causes a serine- (Ser) to glycine (Gly) substitution and significantly increased dopamine-binding affinity.^40^ Polymorphism of the D3 receptor gene has been reported to be associated with the occurrence of impulse control disorders in general,^27,41^ and with impulsive behaviour in PD patients in particular.^42^ To our knowledge, the influence of the D3 receptor gene polymorphism on the risk to develop hypomania in PD patients remains unclear.

In this study, we scrutinised the (I) association of impulsive and hypomanic traits relating to saccadic performance in PD and (II) questioned whether different dopaminergic (D2, D3) gene polymorphisms have an influence on this association. In the last step we (3) conducted a mediation analysis, to question, if clinical parameters or brain connectivity have a relevant influence on the association of saccadic performance onto hypomanic or impulsive behaviour integrating information of the individual genotypes.

## Results

### Clinical and behavioural assessment

**Fig. 1:**
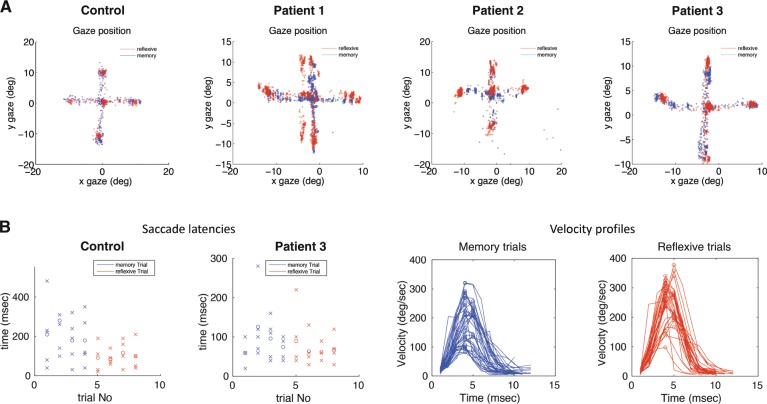
Saccade characteristics in Parkinson’s disease. **a** The exemplary raw gaze positions of one control and three patients are shown. Gaze during reflexive trials is always presented in red, gaze during memory trials is shown in blue. Each dot represents a single eye position measurement. Data are presented in visual angle space (degrees), centred around central fixation at coordinates (0,0). Visual targets were presented at a distance of 10° from the centre in either the vertical or horizontal direction. **b** Data from one control (left) and patient 3 (right) are presented in more detail with saccade latencies (in ms) during reflexive (red) and memory (blue) trials; additionally scheduled for patient 3 are velocity profiles (deg/s over ms) during each trial type. While patients show large differences in precision and the overall eye movement pattern, gaze during memory trials (blue) appears to more often land in between target and central fixation spot than during reflexive trials (red) (**a**). Similarly, latencies show slight differences between memory and reflexive trials in patient 3 (**b**). More prominently, saccadic velocity profiles show saccades with lower peak velocity in memory trials (blue) than in reflexive trials (red), indicating saccades more often stopping short during the memory task.

**Fig. 2:**
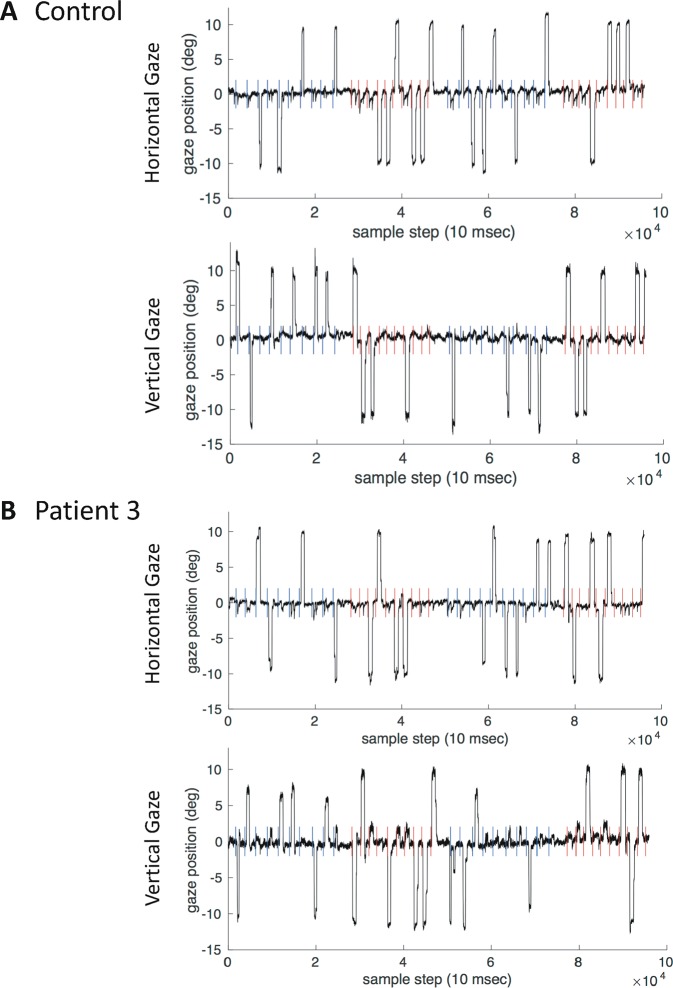
Vertical and horizontal traces. Vertical and horizontal eye position traces (in degree of visual angle) are plotted over time in steps of 10 ms for a healthy control (**a**) and a patient (**b**). In each time series, the respective trial's trigger to initiate a saccade to the previously shown target location (i.e., turning off of the fixation point) is indicated per trial as a coloured vertical line, coloured, according to each trial's stimulus condition, in red (reflexive) or blue (memory).

We scrutinised the association of impulsive and hypomanic traits relating to saccadic performance in PD; all key results are summarised in Table 1 and raw gaze positions of the three patients and one control are visualised in Figs. 1 and 2. The following factors were included in the analysis as saccade parameters: (I) duration, (II) latency, (III) gain and (IV) amplitude.

**Table 1:** Overview of the key results.

Patients		All (*n* = 33)	Correlation with HPS		
VGS		Duration	*r* = 0.50; *p* = 0.003		
		Gain	n.s.		
		Latency	n.s.		
		Amplitude	n.s.		
MGS		Duration	*r* = 0.49; *p* = 0.004		
		Gain	n.s.		
		Latency	n.s.		
		Amplitude	n.s.		
		DRD2+ (*n* = 12)		DRD2– (*n* = 21)	
		Mean (SE)	Correlation with HPS	Mean (SE)	Correlation with HPS
VGS	Duration	92.14 (SE: 1.14)	n.s.	92.1 (SE: 0.94)	*r* = 0.49; *p* = 0.024
	Gain	0.79 (SE: 0.04)	n.s.	0.79 (SE: 0.02)	n.s.
	Latency	514.9 (SE: 12.2)	*r* = 0.61; *p* = 0.036	506.3 (SE: 7.2)	n.s.
	Amplitude	5.097 (SE: 0.32)	n.s.	5.557 (SE: 0.38)	n.s.
MGS	Duration	86.98 (SE: 1.60)	n.s.	89.7 (SE: 1.06)	*r* = 0.47; *p* = 0.033
	Gain	0.60 (SE: 0.007)	*r* = 0.72; p = 0.009	0.66 (SE: 0.03)	n.s.
	Latency	511.2 (SE: 18.39)	n.s.	529.6 (SE: 8.72)	n.s.
	Amplitude	3.923 (SE: 0.30)	n.s.	4.377 (SE: 0.32)	n.s.
		DRD3+ (*n* = 14)		DRD3− (*n* = 19)	
		Mean (SE)	Correlation with HPS	Mean (SE)	Correlation with HPS
VGS	Duration	92.62 (SE: 1.07)	n.s.	91.75 (0.98)	*r* = 0.62; *p* = 0.005
	Gain	0.74 (SE: 0.03)*	n.s.	0.8274 (SE:0.02)*	n.s.
	Latency	511.7 (SE: 9.9)	n.s.	507.7 (SE: 8.4)	*r* = 0.52; *p* = 0.022
	Amplitude	5.243 (SE: 0.42)	n.s.	5.497 (SE: 0.35)	*r* = 0.62; *p* = 0.005
MGS	Duration	89.97 (SE: 1.31)	n.s.	87.77 (SE: 1.22)	*r* = 0.55; *p* = 0.015
	Gain	0.65 (SE: 0.03)	*r* = 0.63; *p* = 0.016	0.63 (SE: 0.04)	n.s.
	Latency	545.3 (SE: 11.45)*	n.s.	506.4 (SE: 11.22)*	n.s.
	Amplitude	4.214 (SE: 0.42)	n.s.	4.21 (SE: 0.28)	*r* = 0.61; *p* = 0.006
Controls		All (*n* = 14)			
		Mean (SE)			
VGS	Duration	97.49 (SE: 0.83)			
	Gain	0.74 (SE: 0.05)			
	Latency	514.82 (16.49)			
	Amplitude	6.01 (SE: 0.57)			
MGS	Duration	96.66 (SE: 1.22)			
	Gain	0.65 (SE: 0.07)			
	Latency	519.55 (SE: 12.62)			
	Amplitude	5.15 (SE: 0.56)			

*Significant at *p* < 0.05

*Patients*: Visualised are the key results of the regression analysis between the visually guided (VGS) and memory-guided saccades (MGS) and the hypomania scale (HPS). The upper table shows results of the whole parkinsonian group. In the lower table regression analysis is subdivided into the individual genotypes [D2 receptor: (+) risk variant; (−) wild type; D3 receptor (+) risk variant; (−) wild type]. We additionally integrated the individual group sizes (*n*) for better interpretation

*Controls*: For better interpretation of results we present individual saccade parameters of duration, gain, latency and amplitude also for the healthy controls in both VGS and MGS conditions

In the first step we performed a group comparison of saccade parameters between patients and controls. Here, we found significant differences between both groups for the duration of VGS with lower values in the patients’ group (*t* = 4.53; d*f* = 46; *p* < 0.0001). Lower values in the saccade duration of the patients were also present in the comparison of MGS between patients and controls (*t* = 5.14; d*f* = 46; *p* < 0.0001) and for the parameter amplitude (*p* < 0.03) in the Mann–Whitney *U* test, which was chosen due to the nonparametric distribution of this saccade parameter.

Multiple regression analysis was then consecutively performed including MGS [*R* = 0.55; *F* (4; 28) = 3.0; *p* = 0.036] or VGS [*R* = 0.55; *F* (4; 28) = 3.0; *p* = 0.033]; both analyses revealed a significant association of HPS measured by the HPS questionnaire (relating to an association with schizotypy, obsessive compulsivity and emotional instability) by the four saccade parameters (see Supplementary Fig. 2); no significance was found for the impulsivity scale (BIS-11).

In the next step we performed a linear regression analysis for each of the four factors: (i) duration, (ii) latency, (iii) gain and (iv) amplitude in both paradigms (MGS and VGS) including the individual genotypes (D2 and D3).

Interestingly, we found for the parameter (i) duration in the VGS and MGS paradigm a significant association of the HPS for the wild-type B1− [VGS: *R* = 0.62; *F* (1,17) = 10.35; *p* = 0.005; MGS: 0.55; *F* = (1,17) = 7.26; *p* = 0.015; see Supplementary Fig. 3], but not for the risk type B1+.

This was the same for the D2 receptor polymorphism, with a significant result for the wild-type A1− [VGS: R = 0.49; *F* (1,19) = 6.006; *p* = 0.024]; MGS: *R* = 0.47; *F* (1,19) = 5.26; *p* = 0.033 (see Supplementary Fig. 4), but not for the risk allele (A1+).

In line with this also saccade latency [VGS: *R* = 0.52; *F* (1,17) = 6.36; *p* = 0.022] and the amplitude [MGS: *R* = 0.61; *F* (1,17) = 9,878; *p* = 0.006; VGS: *R* = 0.62; *F* (1,17) = 10.36; *p* = 0.005] revealed to be significant in the B1− group.

The gain predicted the HPS significantly for the at-risk variants B1+ and A1+ [MGS: *R* = 0.63; *F* (1, 12) = 7.86; *p* = 0.016; MGS: *R* = 0.72; *F* (1;10) = 10,433; *p* = 0.009], indicating a different involvement of genotypes in generation of diverse saccades. By observing saccade latency, the A1+ group also showed significance in the association of the HPS for the VGS paradigm [VGS: *R* = 0.61; *F* (1, 10) = 5.89; *p* = 0.036], though with less statistical significance. All results are summarised in Table 1.

In the last step we conducted a mediation analysis, in order to understand the influence of structural changes and clinical parameters onto regression analysis. Connectivity of two main subcortical motoric output stations in saccade initiation were taken into account into mediation analysis^43^: (i) the nigro-caudatal pathway and (ii) the nigro-collicular pathway (see Supplementary Fig. 1). The following behavioural parameters were included in the analysis: disease duration, UPDRS-ON and UPDRS-OFF (the behavioural results are all summarised in Table 2).

**Table 2:** Results of metadata.

*Patients*	
Number	*n* = 33
Gender	m = 19; f = 14
Age (years)	68.12 ± 8.16*
*UPDRS-III*	
ON	22.85 ± 1.96**
OFF	31.21 ± 2.31**
Hoehn and Yahr	2.29 ± 0.09**
Disease duration (years)	6.82 ± 0.76**
Symptom onset right (r); left (l)	*r* = 18, *l* = 15
LEDD	506.1 ± 59.23**
*PD subtype*	
AR	*n* = 18
TD	*n* = 3
MT	*n* = 12
LI (in decile)	+85.95 ± 2.56**
MMSE	28.12 ± 0.36**
BDI-II	10.06 ± 1.25**
BIS-11	58.67 ± 1.35**
HPS	8.91 ± 0.91**
DRD3 B1+/B1−	14/19
DRD2 A1+/A1−	12/21
*Controls*	
Number	*n* = 14
Age	65.07 ± 6.89*

*Values are given as mean ± SD

**Values are given as mean ± S.E.M

All relevant patient’s metadata are summarised

*UPDRS-III* part III of the Unified Parkinson’s Disease Rating Scale, *ON* under dopaminergic medication, *Off* after dopaminergic withdrawal, *LEDD* levodopa equivalent daily dosage, *PD subtype* parkinsonian subtype, *AR* akinetic–rigidic, *TD* tremor-dominant, *MT* mixed type, *LI* laterality index, *MMSE* mini mental state examination, *BDI-II* Beck’s depression inventory, version II, *BIS-11* Barrat impulsiveness scale, version 11, *HPS* hypomanic personality scale, *DRD 3* dopamine receptor, *DRD 2* D2 dopamine receptor

The mediation analysis only revealed a significant indirect effect for the amplitude in the MGS modality for the variable UPDRS-ON [CI: −2.26 to −0.024]. All other analyses revealed to be non-significant.

## Discussion

Despite the clinical heterogeneity of PD patients and the relatively small number of patients, we found an association of saccade parameters with HPS, but not impulsive behaviour, by saccades. The HPS is designed to measure a predisposition in personality structure to develop HPS, whereas the BIS-11 is appropriate for detecting impulsiveness traits in adolescents.^44^ Hypomanic personality trait has an influence on the personality change after subthalamic deep brain stimulation, which deeply influences the quality of life of the individual patients and their caregivers.^45^ Although questionnaires are the gold standard to measure hypomanic traits, saccadic performance might support the pre-therapeutical detection of patients with a hypomanic personality structure before dopamine replacement therapy^18^ or implantation of deep brain stimulation electrodes.^46^ Moreover, our findings elucidate that the oculomotor and reward system are not segregated but show an association. This is interesting in the context of the basic research of basal ganglia loops, as suggested for striato-nigrostriatal loops as shown in the macaque monkey.^47^

It has been shown that saccadic parameters like latency, amplitude and gain are affected in PD, although with quite inconsistent findings.^12^ Part of these heterogeneities certainly stems from the fact that the disease does not solely affect the motor system, but also includes non-motor symptoms,^15,16^ as shown in this study for the HPS scale. An affection of saccade processing predominantly in MGS has often been described for PD [for overview see, e.g., Terao et al.^48^]. Our approach of interleaved gap and delay conditions, thereby allowing a comparison of VGS and MGS,^13^ provides a quick and rather simple testing device for eye movement patterns. We found that mostly saccade duration was associated with the HPS scores; we hypothesise that the duration of the individual saccades might be ‘prolonged’ due to the blockage of neural processing capacity due to a reward hypersensitivity.^49^ This processing capacity differed between the gene variants (wild type; risk type), which might reflect varying receptor availability of the individual groups. That might explain why specifically the saccade duration was associated with increasing HPS scores and not any other saccade parameters. It has, of course, to be considered that in the OFF state, eye movement artefacts can occur more often and might have led to diverse results due to their close relationship to skeletal movement [as shown in the monkey^50^]. Eye movement analyses in patients suffering from PD can reveal elaborate datasets that require a particular focus on the experimental design, as well as a careful data analysis and artefact exclusion strategies,^13,51^ as have been applied in our study.

Both gene polymorphisms (D2 and D3) influenced the outcome in our study. The main influence was found not only for the B1− wild type regarding the duration, latency and amplitude, but also for the A1− variant. Thus, our results are in line with existing studies, but extend the actual knowledge into functional saccadic network processing being biased by dopaminergic gene variants in PD patients.

The A1 allele of the (*ANKK1*) TaqIA (rs1800497) SNP in humans has been associated with reduced striatal dopaminergic D2 receptor availability.^31–33^ A genetic predisposition to lower D2 receptor density increases the susceptibility to neuroleptic/dopaminergic medication or clinical symptoms that are associated with diseases involving dopaminergic pathology, like e.g., PD.^52^ The dopamine D2 gene variant is of particular interest in the development of PD because it is the primary target for the nigrostriatal dopaminergic neurons controlling motor function, which has direct influence on the signalling of the direct and indirect basal ganglia pathways and can pharmacologically be modulated [as shown recently for Rasagilin^53^]. The most frequently studied allele variation of the D3 receptor gene is the Ser9Gly polymorphism (rs6280^39^), which causes a serine- (Ser) to (Gly) substitution and significantly increased dopamine-binding affinity.^40^ The D3 receptor gene encodes for a receptor protein that is also the target site for antipsychotic agents and dopaminergic stimulation, e.g., the dopamine agonist Pramipexol.

The mediation analysis only revealed a significant effect for the motor impairment showing an association between the amplitude in the memory-guided condition and hypomania. The fact that this analysis was only significant for one condition and type of saccade might be caused by the small sample, which is the main pitfall of the study. Another limitation of the study is that we did not analyse anticipatory saccades, but avoided them by using visual detection latency before starting saccade detection. Anticipatory saccades would be, however, of interest to future studies, especially in the analysis of saccadic changes and behavioural traits like HPS.^24^ Last, as you can see in Table 2 we included different parkinsonian subtypes: most patients had an akinetic–rigidic subtype (AR, *n* = 18), a lot of patients a mixed type (MT, *n* = 12) and only 3 patients had a tremor-dominant PD syndrome. It is notable that also the PD subtype might lead to differences in saccadic processing, a question that has to our knowledge scientifically however not yet been answered.

Sub-cortically, saccadic performance is controlled by three main subregions: SC, CD and SN. The SC controls on one hand fixation and saccade initiation and on the other hand accuracy and velocity of saccades. It may thus constitute a good model to study disease-induced changes in the basal ganglia circuitry.^12^ From an anatomical point of view, the SC is modulated by excitatory projections of the frontal and parietal eye fields via two serial inhibitory influences, one resulting from a connection of the CD to SN and the other from the SN to SC, as it has been shown in the monkey.^8^ The inhibition of the nigro-collicular connection is tonically active, thereby preventing excitatory signals from various cortical areas to elicit unlimited eye movements. In contrast, nigro-caudatal inhibition becomes active only occasionally; it removes the tonic inhibition of the SC, thereby allowing other excitatory inputs to evoke monkeys’ eye movements.^43^ Secondary to presenting pauses in activity, which are associated with the onset of saccades, nigral neurons decrease activity well in advance of the onset of saccades. The modulation of the activity of nigral neurons is predictive of an upcoming saccade choice. This implicates that these neurons are involved in cognitive processing leading to movement, as shown in the monkey.^54^ Specifically, CD contributes to the determination of monkeys’ oculomotor outputs by connecting motivational values (e.g., the expectation of reward) to visual information.^2,55^ Nigral neurons and neurons of other basal ganglia nuclei (e.g., the pallidum and the subthalamic nucleus) also likely contribute to the suppression of unwanted saccades.^54^ Although we were able to quantify brain connectivity in both anatomical connections, the nigro-collicular and the nigro-caudatal tract, no influence as a mediator could be found for these anatomical regions, suggesting a different underlying pathophysiology.

Our results, despite their challenging complexity, support and extend these previous reports by providing evidence that (1) D2 and D3 dopaminergic signalling has a remarkable influence on processing of specific saccades; (2) exactly these saccadic parameters are associated with non-motor behavioural changes; (3) besides disease-specific influence (as measured by the medication response) no influence of brain connectivity changes in the nigro-caudatal and nigro-collicular tract could be found, indicating a different underlying anatomical substrate. Importantly, saccades in our experiment were acquired after dopaminergic withdrawal of >12 h, in patients with long-lasting dopamine agonists even >36 h. Thus, we aimed to present effects that were mostly independent from dopamine treatment and might help to detect patients at risk for a hypomanic state before therapeutic intervention. The influence of dopamine therapy in the long term on brain plasticity is not assessable, but has to be regarded in the interpretation of our results. It is also worth mentioning that the study was consciously designed without normal controls; our goal was specifically the exploration of associations between behavioural traits in PD and saccade parameters. We were able to find an association between saccadic changes in PD and hypomanic traits. The question, if these findings differ from healthy controls with similar genetic setup should be part of future studies.

Last, we have to discuss some important limitations of the results presented here:

(1) The sampling rate of 100 Hz. The sampling frequency of an eye-tracking system refers to how many times per second the position of the eyes is registered by the eye tracker.^56^ The higher the sampling frequency, the better the ability to estimate the true path of the eye when it moves.^57–59^ Only sampling rates above 200–300 Hz allow the undistorted recovery of saccadic peak velocity. In our experiment, we used a sampling rate of 100 Hz, which was the default setting for the clinical measurements of the eye tracker; this might lead to an underestimation of saccadic peak velocities in our experiment; this fact has to be regarded during the interpretation of the results. Future experiments should be obtained with higher sampling rates.

(2) Saccadic intrusions. Another limitation, which has to be regarded in the interpretation of the here presented results is that the data were not relieved from saccadic intrusions. Saccadic intrusions are involuntary, conjugate movements, which take the form of an initial fast movement away from the desired eye position and followed after a short duration, by either a return secondary saccade or a drift.^60^ Square wave jerks (usually 0.5–5°) are one subtype of saccadic intrusions, which are a typical feature in PD, that moves the eye from and back to the fixation point with an intersaccadic interval of about 200 ms.^61^ In addition, changes in PD can be found in the smooth pursuit.^62^ Unfortunately, we did not specifically control for saccadic intrusions in our experiment, though we conducted an outlier detection step. We chose two factors to describe the saccades: the ratio between saccade duration and distance, and the time point of the peak velocity after saccade onset. For both, we calculated the mean of all saccades that remained after the denoising procedure and discarded all saccades that were outside one standard deviation of that mean. In this way, all further analyses were based on a saccade pool of a given subject and experiment that was not biased by extreme outliers (see the section “Outlier detection”). Saccadic intrusions still might have influenced the final results; the presented data should therefore be interpreted with caution.

(3) Blinks. Blinks are known to change the kinematic properties of saccades, probably by influencing the saccadic premotor circuit.^63^ The effects of blinks on saccades, which are tightly coupled to latency, support the hypothesis that blinks cause profound spatiotemporal perturbations of the eye movements by interfering with the normal saccade premotor circuits. One limitation of this study is that we did not specifically analyse eye blinks. Instead, we excluded blinks by filtering using a maximal velocity threshold to detect saccades. We can thus not entirely exclude blinks as part of our dataset. Blinks thus may have an effect on the latency values of the saccades measured here. Again, this has to be regarded when interpreting our results.

Despite the limitations of our set-up and therefore the ability to analyse the eye movement data in great detail, we detected an interesting task-dependent pattern that could inform future studies of pathology and comorbidity in PD.

Summing up, saccadic performance is associated with hypomania in PD; individual saccade types are differently modulated by the D2 and D3 gene variants. Further studies are required (especially case–control studies including healthy controls) in order to elucidate the impact of this association. Interesting, however, is in this study, that we could find, that the oculomotor and reward system are not segregated but show an association.

## Methods

### Clinical and behavioural assessment

Next to 14 age-matched controls, 33 patients were included in the experiment with the diagnosis of idiopathic PD according to the UK Brain Bank Criteria.^64^ Measurements were taken from distinct samples. Patients with relevant concomitant neurological diseases were excluded from the study like having signs and symptoms of dementia, past history of stroke or brain surgery. In all patients, the family histories of other neurological and psychiatric diseases were negative. There was also no history of head injury, metabolic diseases, encephalitis and toxin exposure.

The clinical assessment comprised a video acquisition of the Unified Parkinson’s Disease Rating Scale (UPDRS), part III.^65^ Patients were measured in two conditions: (i) an OFF condition with a levodopa withdrawal during >12 h and (ii) an ON condition after intake of standardised soluble l-Dopa (200 mg). We performed a dopaminergic withdrawal >36 h in patients with long-lasting dopamine agonists. Next to the UPDRS, disease duration and Hoehn and Yahr stage,^66^ symptom onset and levodopa equivalent dosage were acquired.^67^ Furthermore, in all patients handedness was tested using the laterality index.^68^ Dementia was excluded using the mini mental state examination.^69^ To assess the psychometric state, we additionally acquired the Beck Depression Inventory, revision [BDI-II^70^], Barret Impulsiveness Scale 11 [BIS-11^71^] and the HPS.^72^

### DNA isolation and SNP genotyping

DNA from buccal swabs or buffy coat was isolated using the QIAamp DNA Blood Mini Kit (Cat No. 51106, Qiagen GmbH, Hilden, Germany) according to the manufacturer’s protocol. Concentration and quality of the DNA were assessed with an UV/vis spectrophotometer (ND-1000, Peqlab GmbH, Erlangen, Germany). For SNP genotyping of rs6280 (D3 receptor), 20 ng of DNA was analysed using allelic discrimination assays (TaqMan SNP Genotyping Assays, Applied Biosystems by Thermo Fisher Scientific Inc., Waltham, MA, USA). SNP genotyping for rs1800497 (*ANKK1*) was performed with 20 ng of DNA in triplicates using allelic discrimination assays (TaqMan SNP Genotyping Assays, Applied Biosystems by Invitrogen). Genotyping polymerase chain reaction (PCR) was performed on a 7900HT Fast Real-Time PCR system (Applied Biosystems) and the data analysed with Sequence Detection Software (SDS) 2.3 (Applied Biosystems). Groups were defined for the D2 receptor according to *ANKK1* protein [A1− (wild type): GG; A1+: AG, AA] and for the D3 receptor [B1− (wild type): TT; B1+: TC, CC].

### Saccade acquisition

The saccades were acquired when patients were in the UPDRS-OFF state (i.e., >12 h after withdrawal of levodopa; >36 h for patients with long-lasting dopamine agonists). All tests were performed in a specialised oculomotoric lab at the University Hospital Cologne, Neurology. Eye tracking was conducted using a PC [HP Elitedesk 800, Flatron, L1750SQ, Windows XP Professional (2002)]. The experimental room was completely dark during the measurements to protect the patients from external acoustical and visual stimuli. A DLP projector (xd1150 dlp Texas instruments) was fixated on the ceiling at the height of 2.13 m and projected the image to the wall in front of the patients on an area of 1.5 m × 1.12 m, with a projector distance of 2.20 m. This set-up resulted in a display with viewing angles −16 to +16° horizontally and −14 to +14° vertically. The patients were sitting on a chair (height of the seat 45 cm) at a viewing distance of 1.77 m. The head was fixated on a chin rest (adjusted to the individual patient's height for comfort), to avoid head movement artefacts during the acquisition of the saccades (Gerald Kann, Magdeburg, Germany).

Video oculography (VOG) allows non-invasive acquisition of eye movement. In our study, we applied VOG of the Merz Company, BioMed Jena GmbH (Jena, Germany). The patients were asked to wear special glasses with in-built cameras on the left and right side. These cameras were aligned to the nose of the patient; a transparent mirror was integrated into the glasses so that the camera could detect the patients’ pupils. The movements of the eyes were detected two-dimensionally (i.e., in the horizontal and vertical plane) by registration of the pupil in temporal steps of 10 ms (100 Hz). Patients were questioned for amblyopia and potential refraction anomaly was compensated by contact lenses (1-Day Acuvue Moist, Johnson & Johnson), because patients were unable to wear glasses under the head-mounted mask of the VOG. No patient suffered from a red/green weakness. For further information on refractory anomalies, see Supplementary Table 1.

Two experimenters performed the acquisition of the saccades and were also explicitly trained to insert contact lenses (Laboratory of Contact Lenses, University Hospital Cologne, Germany). After optimal positioning of the glasses in the VOG and focusing of the camera onto the pupils, the eye tracker was calibrated. During the calibration procedure, nine points were presented sequentially (3 × 3); the patient was asked to fixate the individual points and the experimenter then confirmed each fixation via button press in the eye tracker's software. Following this procedure, experimental testing was conducted. To avoid comprehension problems during the experiment, the whole test was explained and demonstrated by the experimenter. The whole experiment's duration was ~6 min. We used two different paradigms: (1) VGS, and (2) MGS, which will be described in more detail below. Each patient performed one test run and two experimental runs. Every run lasted around ~1.5 min. In the individual runs, 2 blocks each of MGS and VGS (containing 10 trials each) were interleaved with short breaks of 2 s between blocks (transition pause). During the transition pauses, a dot was presented in the centre of the display (0° vertical, 0° horizontal) with its colour differing from the initial fixation point, such as to indicate a switch in trial type from MGS to VGS or vice versa.

**Fig. 3:**
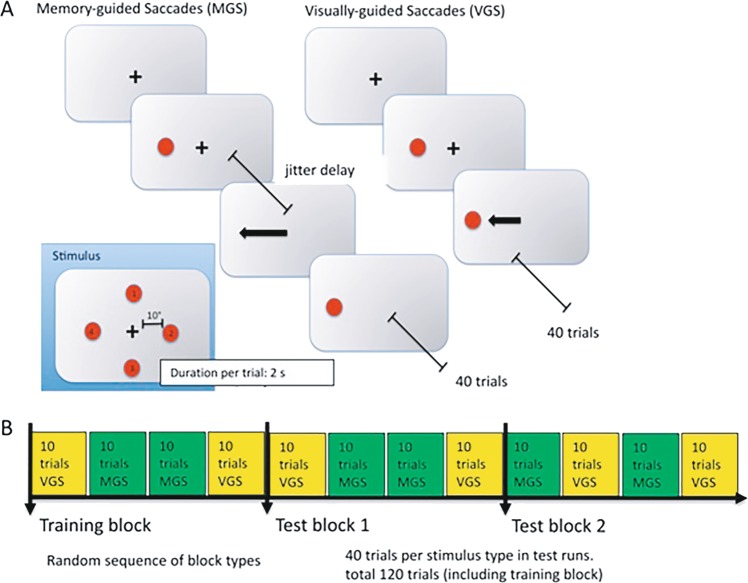
Experimental design of the MGS and VGS. **a** Each experimental run consisted of a sequence of blocks of memory-guided saccade task trials (MGS) and visually guided (VGS) saccade task trials. After a fixation phase (shown as central cross in Fig. 3a), the saccade target (red dot) was presented alongside the fixation target. Saccade targets were placed at one of four possible locations (Fig. 3a inset) with a distance of 10° from central fixation. Participants were asked to saccade to the target only after the offset of the fixation cross. In VGS trials, this offset occurred right away, that is, saccades were allowed right after onset of the saccade target. In MGS trials, the fixation target stayed visible for a variable delay (jitter delay) after offset of the saccade target. That is, patients had to memorise the target's location, and only after a delay, they were allowed to initiate the saccade to that location. Each trial lasted around 1.5 s. Thirty MGS and 30 VGS trials were presented per run. **b** Blocks of MGS- and VGS trials were presented in a randomised sequence, summing up to a total of 240 trials and about 6 min of testing time.

After the first experimental run, the nine-point calibration, as described above, was repeated to correct for potential movement artefacts of the patients (e.g., due to swallowing, turning of the torso or head). The background of the projection area was black, the fixation point during calibration was white, the fixation point during the experiment was white and the stimuli (MGS and VGS) were green. The fixation point during the transition pause was also shown in green. Testing paradigms: (1) for the VGS, patients were asked to fixate on the central black fixation cross. Green target stimuli were then presented randomly in one of four possible positions (10°/0°, −10°/0°, 0°/10° and 0°/−10°, horizontal/vertical), while simultaneously the central fixation point disappeared. This occurred at 900 ms + variable delay (between 0 and 500 ms) after fixation onset. Patients were asked to saccade to the target as fast as possible and maintain fixation until the central fixation point reappeared. The saccade target then stayed visible for the entire trial duration (2000 ms). With two blocks of 10 trials per experimental run, and two runs, each patient conducted 40 VGS trials in total. (2) During the acquisition of the MGS, the patient was asked to fixate on the central black fixation cross. During fixation, a target stimulus was presented 900 ms after fixation onset at a peripheral (10° distance), randomly chosen (one of four possible positions, same as in VGS trials) location and extinguished after 100 ms later. The patients were asked to maintain fixation of the central cross, i.e., to suppress a reflexive saccade to the new stimulus, while remembering its position. The patients were then asked to saccade to the remembered location (without the target being visible) after the central fixation point disappeared. This happened 1300 ms after fixation onset plus a delay, which was randomly chosen in each trial (between 0 and 500 ms). Thus, the saccade target was visible for 100 ms, and the signal to saccade occurred between 300 and 800 ms later. MGS trials lasted for 2500 ms to provide ample time for patients before the next trial started, as indicated by the presentation of the fixation cross. This test was in total repeated 40 times, in an analogous fashion to the VGS experiment. For an illustration of the experimental procedure and the trial sequence, see Fig. 3. The healthy controls were tested in analogous fashion.

### Parsing

Raw eye-tracking data consisted of eye position coordinates in degrees of visual angle on the display area, sampled with 100 Hz; data streams also contained markers at the timepoints of stimulus presentation (fixation start or end, target start or end and custom modification of the eye tracker software by BioMed Jena GmbH). These data were automatically parsed to a Matlab matrix file for further analysis.

Data were displayed as x/y gaze coordinates for a first inspection of general recording quality. Because the eye tracker did not detect saccades automatically, saccades and blinks were detected using Matlab. To this end, we calculated a smoothed velocity profile of the entire dataset with a moving window of three recording steps (i.e., 30 ms). Given the distance of the target to the fixation point being 10°, a maximal saccadic velocity of ~500–600 deg/s can be expected (c.f.‚ main sequence of saccades).^73^ Therefore, blinking artefacts were defined as eye movements surpassing a threshold of 800 deg/s, thereby also allowing for a range of overshoot.

Individual saccades were detected using a threshold of 30 deg/s. Besides, we defined a threshold for a minimum peak velocity of the saccades of 45 deg/s. Furthermore, we excluded every saccade with a duration shorter than 20 ms, because of the limited temporal resolution of our eye tracker (100 Hz), which would not allow detecting the start and end of short saccades with a velocity threshold of 30 deg/s. This step was necessary, as some recordings' noise patterns produced artefacts mimicking such short saccades. All detected saccades, which had a longer duration than 90 ms, were also excluded from further analysis as measurement artefacts, because saccades crossing a distance of 10° can typically last only about 50 ms (according to the main sequence^73^).

### Outlier detection

Individual datasets can contain quite a variety of saccadic patterns; subjects can be distracted and follow a sound, or forget what they were supposed to do and look around the display aimlessly. This is especially true for patients with diseases affecting eye movement, vigilance or attention. To allow a better statistical estimate of the average saccadic pattern within each subject and experiment, we conducted an outlier detection step. Some experiments assure that the attention is not lost by including a button press, when subjects see the stimulus; this however might interfere with the processing of the eye movement due to the higher complexity of the task and herewith affect individual saccade parameters. That is why we chose not to measure attention separately. We chose two factors to describe the saccades: the ratio between saccade duration and distance, and the time point of the peak velocity after saccade onset. For both, we calculated the mean of all saccades that remained after the denoising procedure and discarded all saccades that were outside one standard deviation of that mean. This way, all further analysis was based on a saccade pool of a given subject and experiment that was not biased by extreme outliers.

### Trial time window

Detected saccades were chosen from a time window starting at the onset of the saccade target, and ending after the offset of the saccade target. Given that subjects were to look at the target only after the fixation cross had disappeared, we used two time windows, ‘preFix’ (in which subjects conducted saccades prematurely), ending at fixation offset plus a visual detection latency of 80 ms, and ‘postFix’ (in which subjects were expected to perform a saccade), starting at fixation offset plus 80 ms.

Within the postFix window, we calculated the following parameters for both VGS and MGS conditions: (a) mean latency (in ms), i.e., the duration from fixation offset until the saccade started; (b) mean amplitude; (c) mean duration of the individual saccades (ms). We used separate measures for the first saccade in a trial, and for all saccades during a trial, to account for potential differences in correction saccades based on effects such as error signal processing and heightened attention due to landing errors; (d) gain of the saccades (i.e., the ratio of the actual saccadic amplitude, from fixation to saccade landing, to the intended amplitude, i.e., from fixation to target). This value is typically in the range of 0.9, i.e., healthy subjects' saccades tend to undershoot the target location by about 10%, but it has been shown to be substantially smaller in PD (cf.^12^).

### Acquisition and processing of structural MR images

All magnetic resonance data have been obtained on a Siemens 3 T PRISMA scanner using a 64-channel head coil. The high-resolution T1-weighted images were acquired by applying a 3D MPRAGE sequence (TR = 2300 ms, TE = 2.32 ms, ES = 7.2 ms, FA = 8°, FOV = 230 mm × 230 mm, isotropic pixel resolution of 0.9 mm and slice thickness of 0.9 mm, 192 slices). The T2-weighted data were acquired using a SPACE sequence (TR = 3200 ms, TE = 460 ms, ES = 4.38 ms, FOV = 256 mm × 240 mm, isotropic pixel resolution of 0.8 mm and slice thickness of 0.8 mm, 224 slices). By applying a spin-echo planar imaging sequence, the diffusion-weighted volumes were acquired for 90 unique gradient directions with a weighting factor of 3000 s/mm^2^ (*b* value) (TR = 16.8 s, TE = 82 ms, ES = 0.74 ms, FOV = 220 mm × 220 mm, isotropic pixel resolution of 1.72 mm and slice thickness of 1.7 mm, 90 slices, AP phase encoding). In addition, images without any diffusion weighting were acquired at the beginning of each block of nine diffusion-weighted volumes, as well as at the beginning and the end of the measurement. Since the applied EPI sequence is highly influenced by susceptibility distortions, especially at locations of tissue borders, an additional set of eight non-diffusion-weighted volumes with a reversed-phase encoding direction (posterior to anterior) was obtained.

The preprocessing of all MR data was performed using the Oxford FSL toolbox (v5.0.8; https://fsl.fmrib.ox.ac.uk/fsl/fslwiki/) and the Freesurfer software package (v5.3.0; https://surfer.nmr.mgh.harvard.edu). Initially the T1-weighted images were preprocessed for brain extraction using the Freesurfer reconstruction pipeline (autorecon1). For enhancing the resulting brain estimate, this step additionally implies a prior intensity inhomogeneity correction and a linear transformation into the Talairach space. Thus, the resulting brain mask had to be transformed back into the native anatomical T1 space, applying a linear registration. To further apply the generated brain mask onto the T2-weighted anatomical image, T1- and T2-weighted data were linearly aligned using a rigid-body registration with 7 degrees of freedom in FSL-FLIRT. Afterwards all anatomical images were spatially normalised by an affine registration with 12 degrees of freedom into the MNI standard space. For addressing the problem of susceptibility-induced distortions, the first step in processing the diffusion-weighted data was to correct for those distortions using FSL-TOPUP. Therefore, non-diffusion-weighted volumes acquired using the anterior–posterior and the posterior–anterior phase encoding direction had to be merged into a single 4D image. After averaging the distortion-corrected non-diffusion-weighted volumes, this image was linearly registered into the anatomical space based on the T2-weighted image. To avoid inaccurate estimates of brain tissue in diffusion-weighted data, the brain mask generated from the T1-weighted volume was accordingly aligned into the diffusion space and applied to the non-diffusion-weighted image. Based on the previously generated distortion estimates (FSL-TOPUP), the 4D image data including the diffusion- and non-diffusion-weighted imaging data acquired using the anterior–posterior phase encoding were passed into FSL-Eddy and corrected for eddy currents, susceptibility-induced distortions and motion. For improving the anatomical accuracy in white matter tractography, the corrected 4D data were interpolated to 1 mm × 1 mm × 1 mm voxel resolution. All subsequent processing steps, like tensor reconstruction (FSL-DTIFIT) and the fibre model generation (FSL-BEDPOSTX) were accordingly performed on these interpolated images.

Further preprocessing was performed with FSL v5.0.4. A reorientation of 3D–T1-weighted images to the sagittal plane through the anterior and posterior commissures was performed. This 3D–T1-weighted image as individual structural space of each subject was used as a high-resolution image for further analysis. Individual T1 images were non-linearly registered to the MNI-1mm space by the application of FNIRT; the resulting warp field was then inverted (FSL-INVWARP) and co-registered to the individual B0 image by applying a rigid-body transformation (=6 DOF) for transformation to diffusion space. After nonlinear registration of T1- and T2-weighted images into standard space, masks were traced singularly in MNI-152-1mm space with fslview (http://fsl.fmrib.ox.ac.uk/fsl/fslview/) by the implementation of FA and V1 maps, to get one ‘template’ mask per subregion.

The main subcortical motoric output stations in saccade initiation were chosen for brain connectivity measures^43^: SN, CD and SC. Mask location was chosen based upon different anatomical atlases and segmentation protocols were applied to get a distinguished anatomical reliability per mask.

### Substantia nigra

For the outlining procedure of the SN the following protocol was chosen according to the MRI atlas of Naidich et al.:^74^ nigral borders were defined on T2-weighted images^75^; the masks was initially labelled in the axial planes and controlled in coronal and sagittal planes. The outlining procedure included both (1) the compact part and (2) the reticular part due to methodological limitations of the delineation procedure resulting from the high iron content of the MR images. Please note that the ventral tegmental area and the medial lemniscus could not safely be excluded in this labelling process. Bordering structures were anteriorly the cerebral peduncles, ventro-medially the red nucleus and latero-dorsally the nucleus subthalamicus. Nigral masks were labelled slightly ‘bigger’ than those visually seen on MR images to ensure the delineation of the entire structure. To reduce variability and enhance specificity, probabilistic tractography to the putamen was run initially; the resulting ‘seed_to_target’ masks were then chosen for further analysis to ensure specificity for nigro-putaminal connections despite adjoining the main fibre bundles like the cortico-spinal tract and rubro-thalamic tract.

### Superior colliculus

Starting with a coronary view, the SC appears more hypodense than the inferior colliculus, which is located more caudally. In the axial plane the medial geniculate nucleus defined the lateral, the hyperdense periaqueductal grey defined the ventro-medial boundaries. A right-angled axis was drawn from the medial geniculate nucleus to the periaqueductal grey to set the rostral boundary. The cisterna ambiens was defined as posterior boundary.

### Caudate nucleus

For the mask protocol, see Pelzer et al.^76^.

The FDT toolbox of FSL-Software version 5.0.4 was applied for tractography (https://fsl.fmrib.ox.ac.uk/fsl/fslwiki/). We calculated probability distributions between seed- and target regions by the application of PROBTRACKX. The number of samples was *P* = 5000, the number of steps *S* = 2000 with a step length of 0.5 and a curvature threshold of *c* = 0.2. All target regions were defined as waypoint masks.

Connections were analysed for the nigro-collicular as well as for the nigro-caudatal pathways, each in both directions. The tractography approach underlying PROBTRACKX generates sample ‘streamlines’, or trajectories, to build up a distribution on the location of a connection;^77,78^ this correlates with the anatomical distribution of fibres. Here, we were interested in the probability of the tract starting at A and passing through B. In probability terms, this is equal to the number of streamlines from A that reach B (call this K), divided by the total number of streamlines from A (call this N). To get K, we run PROBTRACKX with A as a seed and B as waypoint; then K was stored in a text file called waytotal. To get N, we multiplied the number of voxels in A by the number of samples per voxel (default = 5000)

$$\phi \left( {A + B} \right) = \frac{K}{N},$$

with *K* = waytotal and *N* = voxelseed* 5000 (default).

Connectivity values for the SN-SC and SN-CD connection were determined by the arithmetic mean of ‘forward’ and ‘backward’ tractography.

### Statistical analysis

In the first step we compared saccade parameters (I) duration, (II) latency, (III) amplitude and (IV) gain between patients and controls.

In the second analysis step we conducted a multiple regression analysis in SPSS® 25, including (I) duration, (II) latency, (III) gain and (IV) amplitude as predictors and HPS and BIS-11 as dependent variables. In a second step we then performed a linear regression analysis for each individual predictor (duration OR latency OR gain OR amplitude) with the dependent variable for the different gene variants A1−/A1+ and B1−/B1+. Regarding their significance, the results derived from all regression analyses were then automatically tested in SPSS Version 25 with the implemented two-sided *t* test; a value of *p* < 0.05 was defined to be significant. The resulting significant regression analyses were then consecutively transferred into a mediation analysis [model 4^79^], in order to determine how the regression analysis might be influenced by the mediator variables (disease duration, UPDRS-ON, UPDRS-OFF, nigro-collicular connectivity and nigro-caudatal connectivity).

### Ethics statement

The study was performed with the understanding and written consent of all participants and followed the declaration of Helsinki. The study has been approved by the local ethics committee in Cologne (12-268).

### Reporting summary

Further information on research design is available in the Nature Research Reporting Summary linked to this article.

## Supplementary information

Supplementary Information

Reporting Summary

## Data Availability

The data that support the findings of this study are available from the corresponding author upon reasonable request.

## Code availability

Code for preprocessing of structural data can be accessed at https://github.sf.mpg.de/TNC/kfo2-parkinson.git (Makefile.koeln-probands.struct2_fs). Code for preprocessing of diffusion-weighted data can be accessed at https://github.sf.mpg.de/TNC/kfo2-parkinson.git (Makefile.koeln_prisma.dwi). Code for registration of structural datasets to diffusion-weighted data can accessed at https://github.sf.mpg.de/TNC/kfo2-parkinson.git (Preproc_Eyepark). Code for processing steps to run diffusion tractography can be accessed at https://github.sf.mpg.de/TNC/kfo2-parkinson.git (EYE_ANAT_TRAKTOGRAPHY). Please see Supplementary Fig. 5 for the flow charts.
