# Supplementary material for: Hypomania and saccadic changes in Parkinson’s disease: influence of D2 and D3 dopaminergic signalling
Source: NPJ Parkinsons Dis. 2020 Jan 17;6:5. doi: 10.1038/s41531-019-0107-3 (PMC6969176; doi:10.1038/s41531-019-0107-3)

# Supplementary

## Supplementary Table 1:

### Refractory abnormalities the parkinsonian patients

| | Diopter | | Correction (dpt) | | Eye disease | Add. information |
|---|---|---|---|---|---|---|
| No° | right | left | right | left | | |
| 1 | 0 | +0,5 | - | - | Cataract | |
| 2 | 0 | 0 | - | - | age farsightedness | just reading glasses |
| 3 | +2,25 | +2,25 | +2,0 | +2,0 | - | |
| 4 | +1,0 | +1,5 | +1,0 | +1,0 | - | |
| 5 | +1,5 | +1,5 | +1,0 | +1,0 | - | |
| 6 | +1,5 | +1,5 | +1,0 | +1,0 | - | |
| 7 | -1,5 | -1,25 | -1,0 | -1,0 | Cataract | |
| 8 | 0 | +1,0 | - | - | Disc drusen | no lenses tolerated |
| 9 | +0,25 | +0,75 | - | - | Cataract | |
| 10 | -1,0 | +0.25 | -1.0 | - | Cataract | |
| 11 | -0.5 | +1,5 | - | +1,0 | Cataract | |
| 12 | +0,5 | +1,0 | - | - | - | no lenses tolerated |
| 13 | +2,5 | +2,5 | +2,0 | +2,0 | - | |
| 14 | +1,5 | +0,5 | +1,0 | - | - | |
| 15 | +4,0 | +4,0 | +4,0 | +4,0 | - | |
| 16 | -1,75 | -2,0 | -2,0 | -2,0 | - | |
| 17 | -1,75 | -1,75 | -2,0 | -2,0 | after eyelid op | |
| 18 | +1,75 | -1,25 | +2,0 | -1,0 | - | |
| 19 | -0,5 | -0,25 | - | - | Cataract | |
| 20 | -7,0 | -6,5 | -6,0 | -6,0 | - | |
| 21 | -1,0 | -2,75 | - | -3,0 | Cataract | no lense on the right eye tolerated |
| 22 | +1,5 | +1,25 | +1,0 | +1,0 | - | dry eyes |
| 23 | +2,75 | +2,75 | +3,0 | +3,0 | - | - |
| 24 | +0,5 | +0,5 | - | - | - | - |
| 25 | 0 | 0 | - | - | Cataract, age farsightedness | just reading glasses |
| 26 | 0 | 0 | - | - | Cataract, age farsightedness | just reading glasses |
| 27 | -0,25 | 0 | - | - | Cataract | - |
| 28 | +1,5 | +1,5 | +1,0 | +1,0 | - | - |
| 29 | -1,75 | -1,75 | -2,0 | -2,0 | - | - |
| 30 | 0 | 0 | - | - | age farsightedness | just reading glasses |
| 31 | 0 | 0 | - | - | trauma of the left eye, age farsightedness | just reading glasses |
| 32 | 0 | 0 | - | - | age farsightedness | just reading glasses |
| 33 | 0 | 0 | - | - | age farsightedness | just reading glasses |

# Supplementary Figure 1:

## Tractography of the nigro-caudatal and nigro-collicular tract on the left side

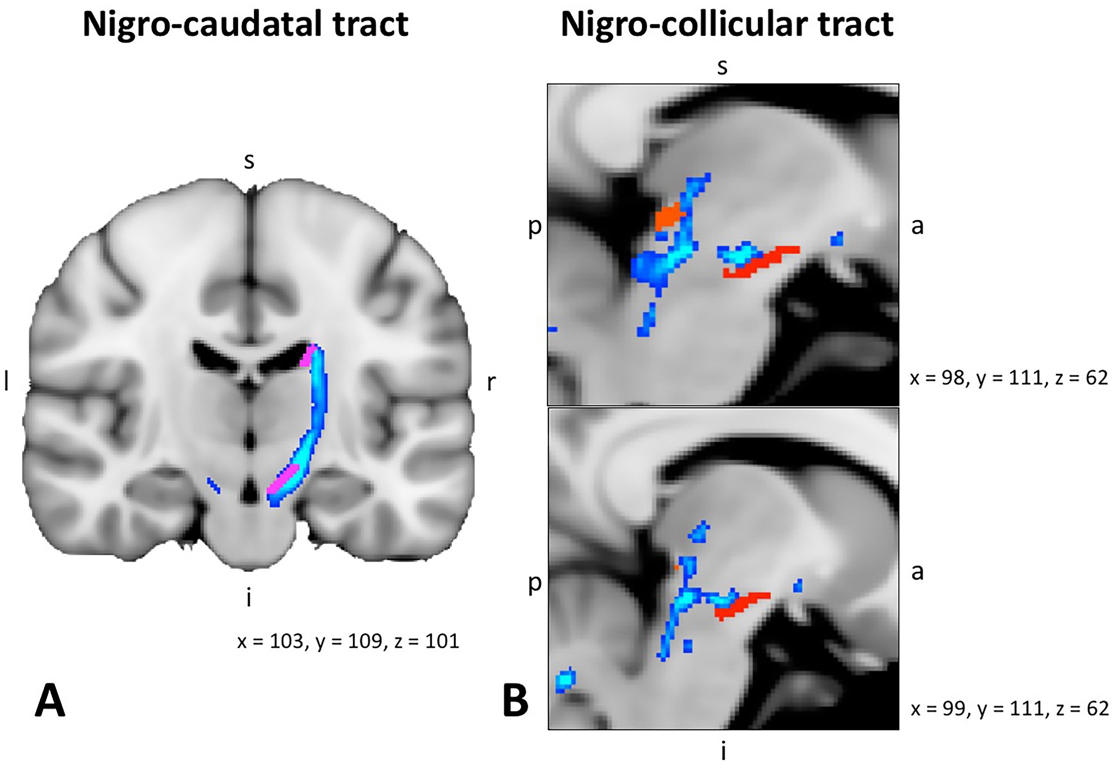


**A** Coronal view of projections from the SN to the CD at x = 103; y =109 and z =101. *Pink= Masks of SN (inferior) and CD (superior); blue-lightblue= projections between these to structures.*

**B** Sagittal view projections from the SC (posterior-superior) to the SN (anterior-inferior) at level x =98 - 99; y =111; z = 62. *Red= Mask of SN* (anterior-inferior) *and SC (posterior-superior); blue-lightblue= projections between these two structures.*

## Supplementary Figure 2:

**Linear regression analysis of the HPS in the whole parkinsonian group**

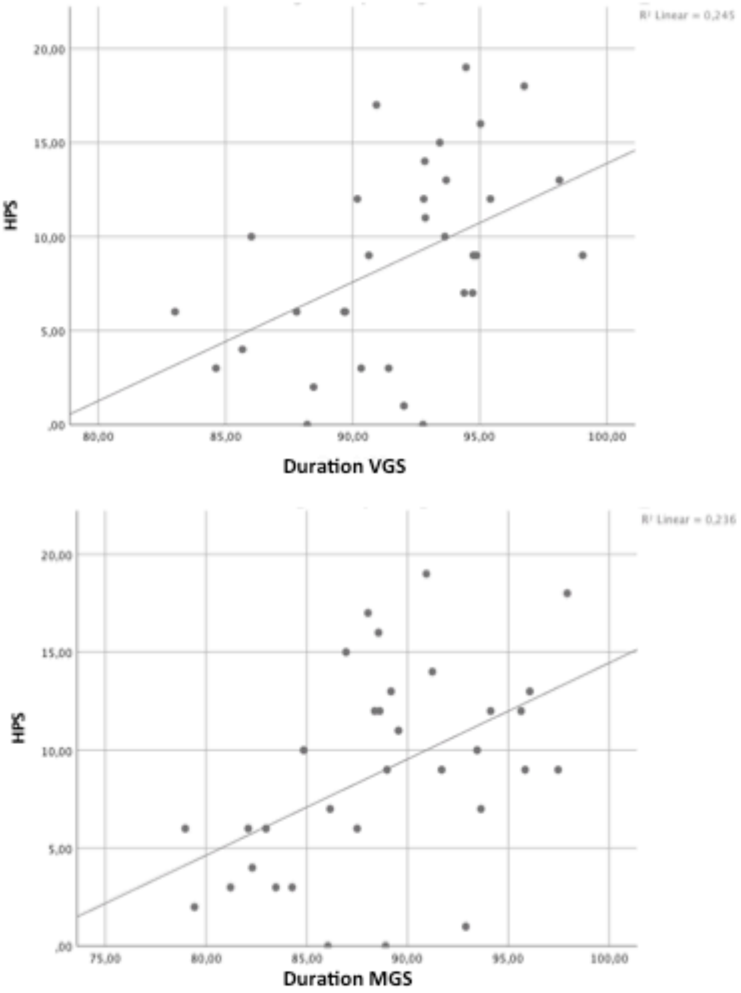


Both, the visually (top) and memory-guided (bottom) saccades are associated with the scores in the HPS reliably.

# Supplementary Figure 3:

**Linear regression analysis of the HPS in the DRD3- group**

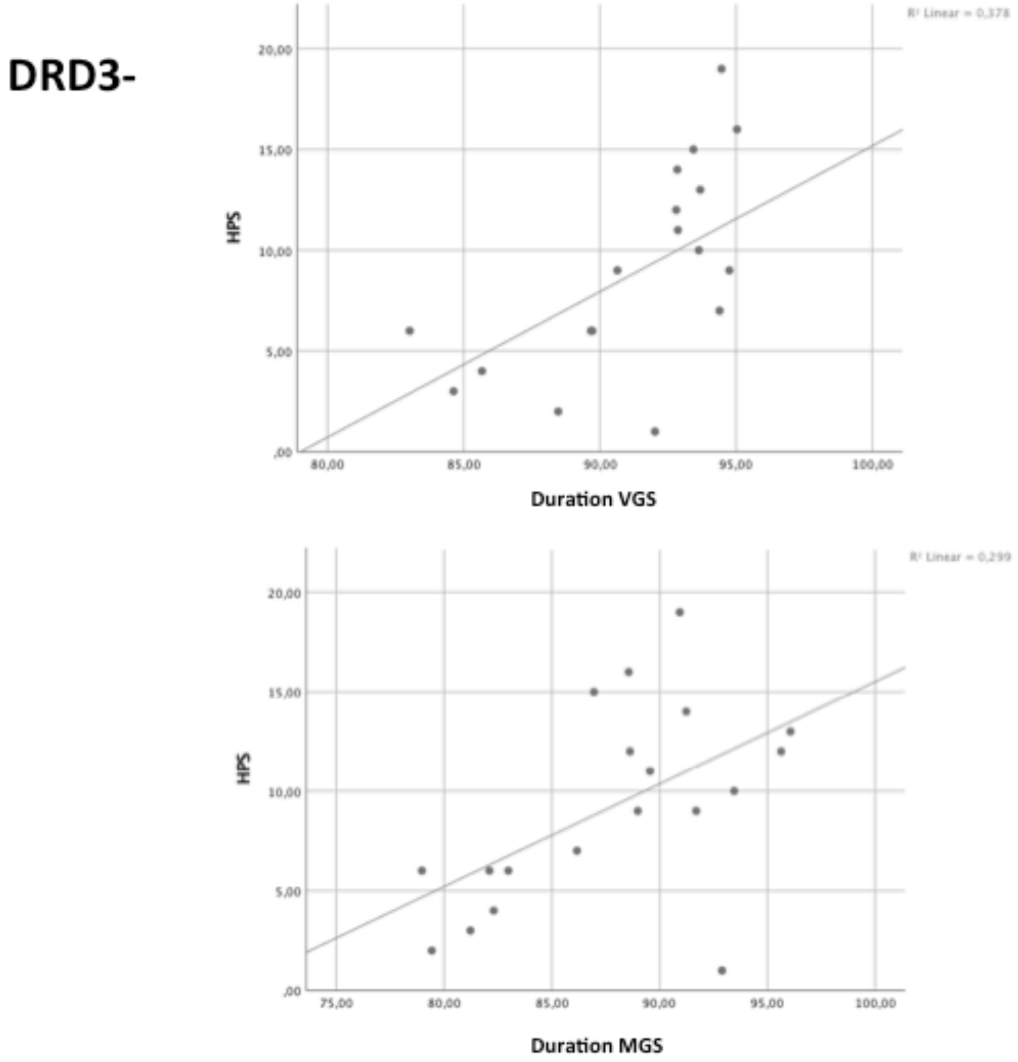


Both, the visually (top) and memory-guided (bottom) saccades are associated with the scores in the HPS reliably.

## Supplementary Figure 4:

**Linear regression analysis of the HPS in the DRD2- group**

**DRD2-**

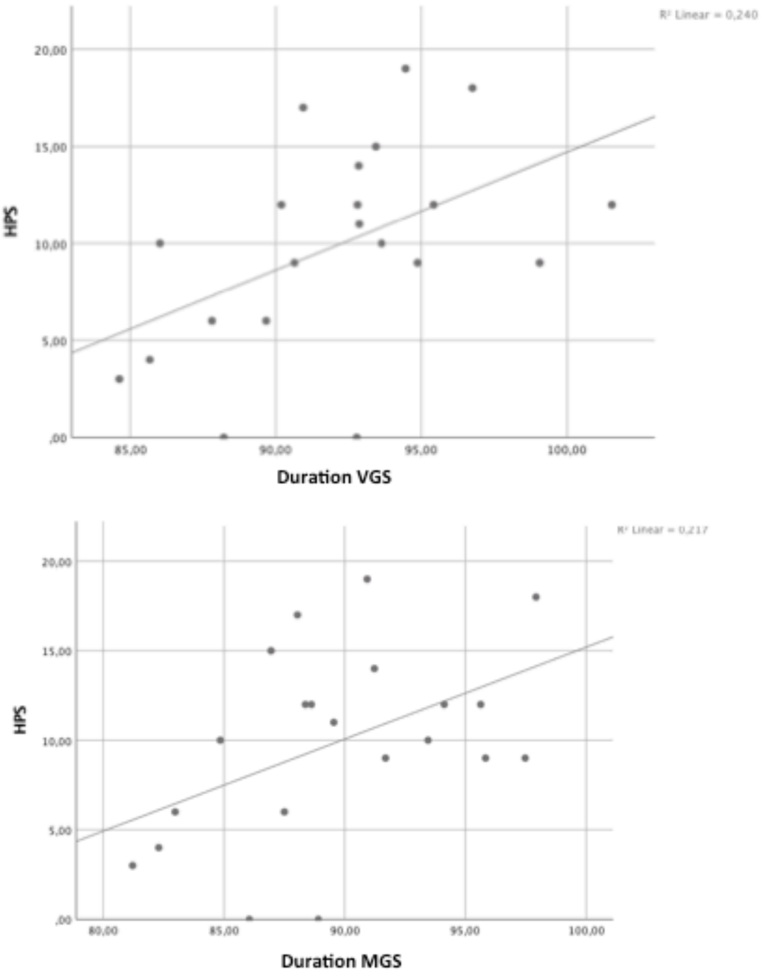


Both, the visually (top) and memory-guided (bottom) saccades are associated with the scores in the HPS reliably.

## Supplementary Figure 5:

### a) Preprocessing of structural data

(see https://github.sf.mpg.de/TNC/kfo2-parkinson.git; Makefile.koeln-probands.struct2_fs)

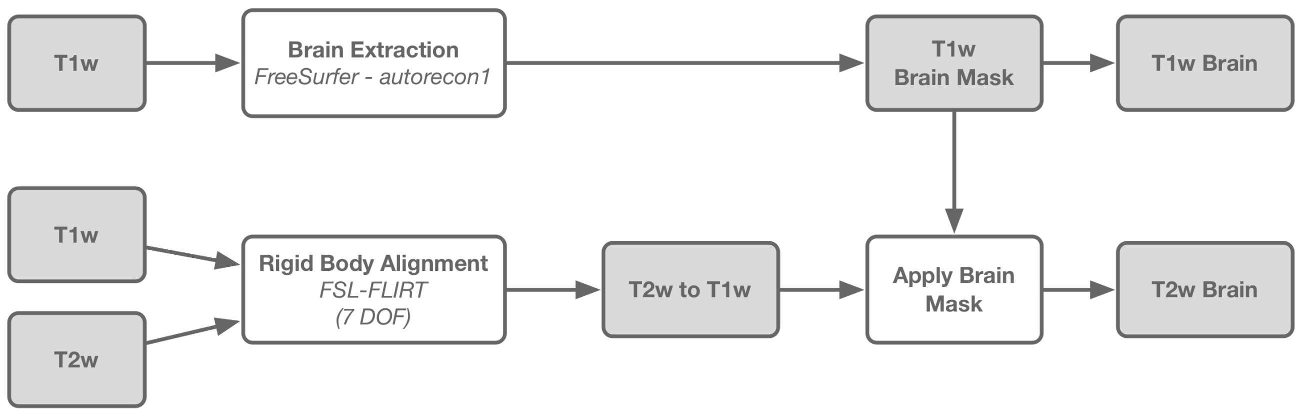


### b) Preprocessing of diffusion weighted data

(see https://github.sf.mpg.de/TNC/kfo2-parkinson.git; Makefile.koeln_prisma.dwi)

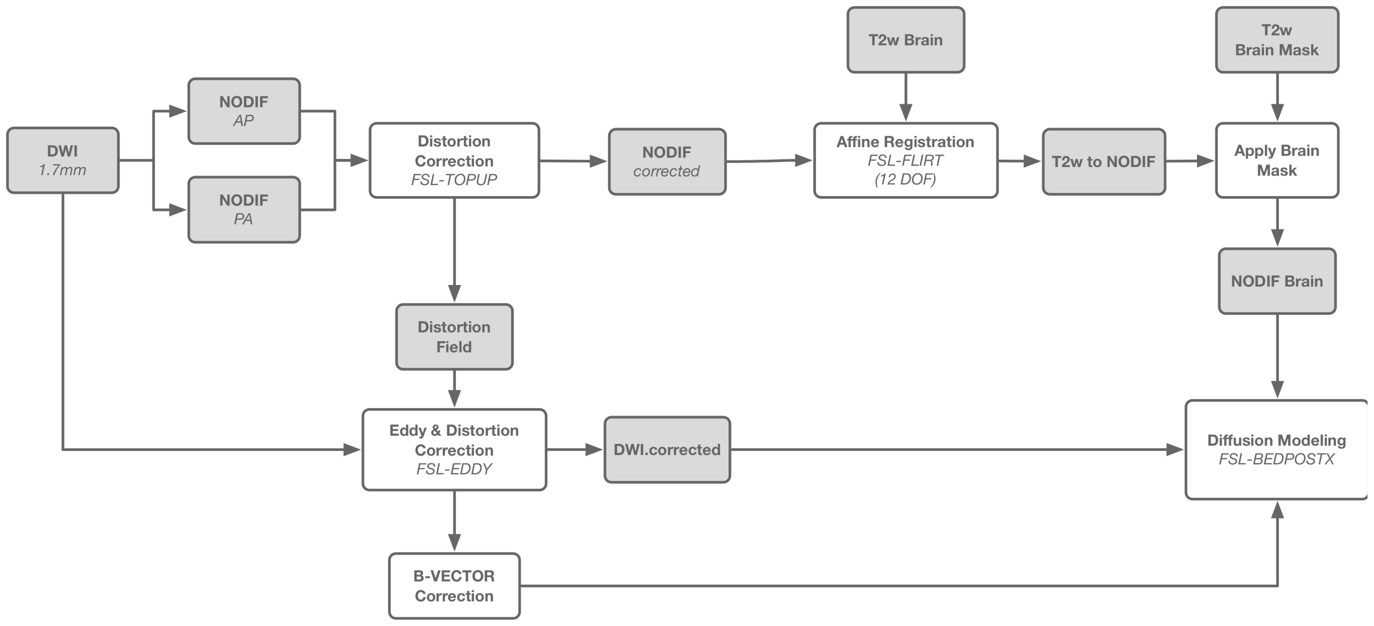

**c) Registration of structural datasets to diffusion weighted data**
(see https://github.sf.mpg.de/TNC/kfo2-parkinson.git;
Preproc_Eyepark)

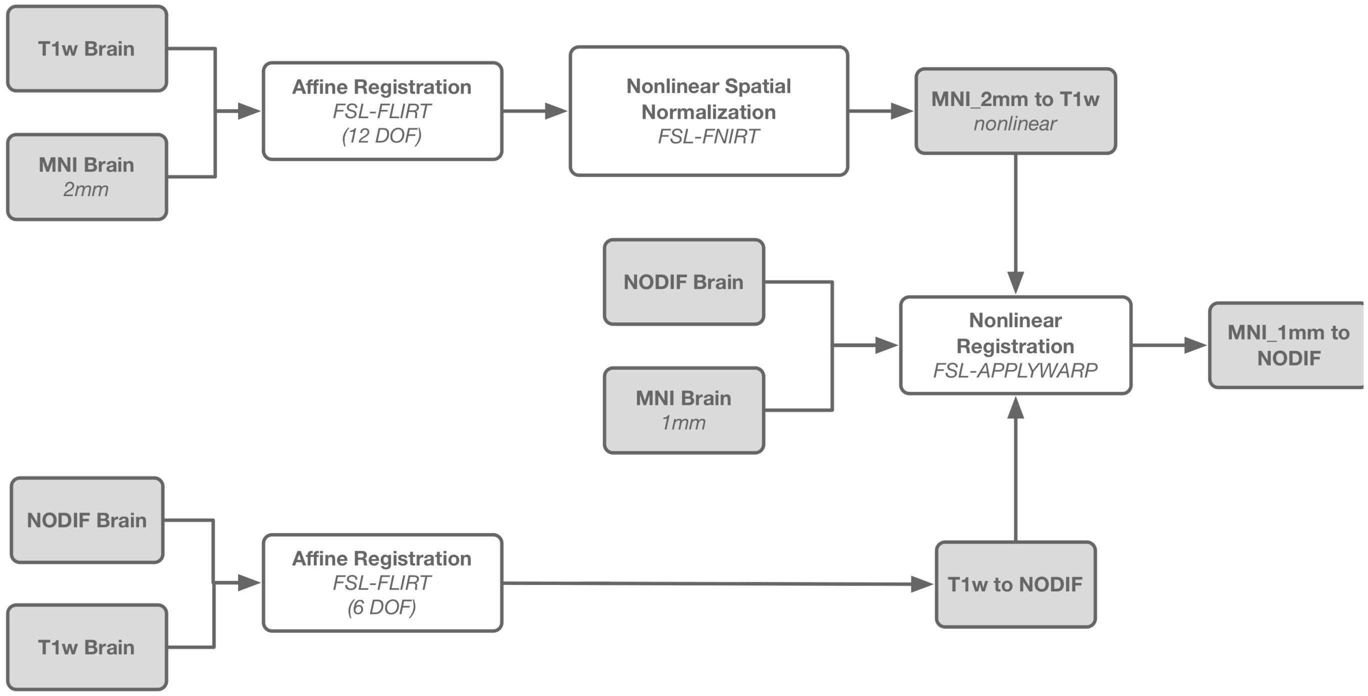


**d) Processing steps to run diffusion tractography**
(see https://github.sf.mpg.de/TNC/kfo2-parkinson.git;
EYE_ANAT_TRAKTOGRAPHY)

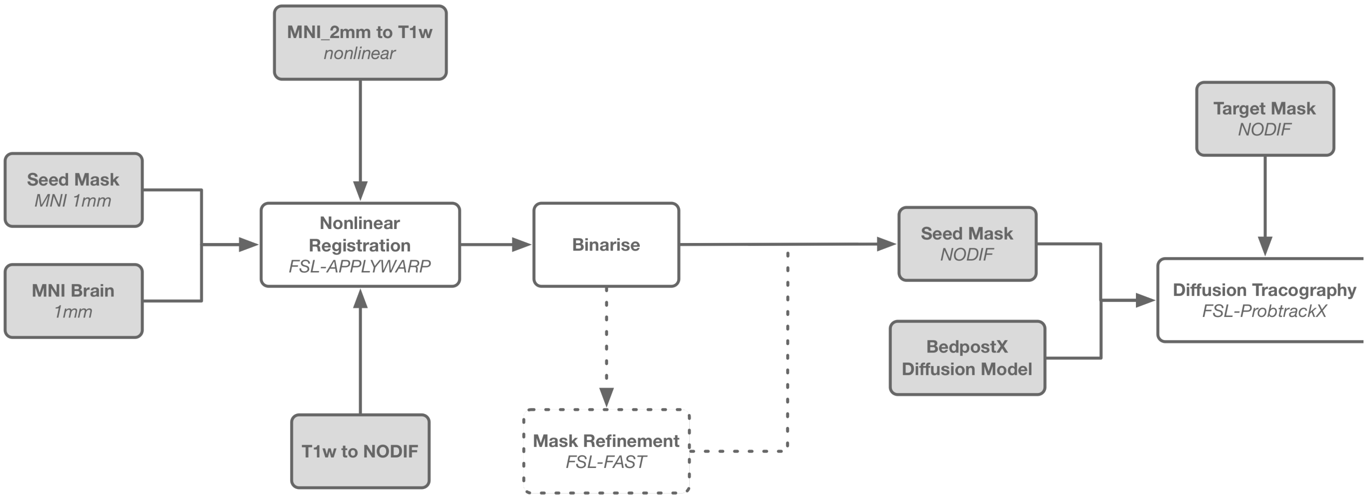

Supplement: Supplementary file 1 — Supplementary Information [file 41531_2019_107_MOESM1_ESM.pdf]
